# Editorial: Focus on Systemic Lupus Erythematosus

**DOI:** 10.3389/fimmu.2016.00400

**Published:** 2016-10-03

**Authors:** James Harris, Eric F. Morand

**Affiliations:** ^1^Department of Medicine, School of Clinical Sciences at Monash Health, Monash University, Clayton, VIC, Australia

**Keywords:** SLE, inflammation, autoimmune diseases, autoantibodies, cytokines, cell death

**The Editorial on the Research Topic**

**Focus on Systemic Lupus Erythematosus**

Systemic lupus erythematosus (SLE) is a serious systemic autoimmune disease. In the absence of specific targeted therapies, the majority of patients suffer inadequate disease control: a 20-year-old diagnosed with SLE has up to a 10% chance of death before turning 40. SLE has long been considered the archetypical autoimmune disease, involving innate and adaptive mechanisms of inflammation and the generation of autoantibodies. A combination of genetic predisposition and environmental triggers leads to activation of the innate immune system. This results in the release of inflammatory mediators, including cytokines, which activate both innate and adaptive immune responses. Autoantibodies bind nucleic acids or cellular debris to form immune complexes, which further activate innate immune cells through Fc and toll-like receptors. This positive feedback loop eventually leads to systemic inflammation and tissue damage, which feeds back to further potentiate immune responses.

The apparent simplicity of this model belies the complexity seen by the physician. SLE presents with a broad range of symptoms and manifestations; some patients might develop renal disease, others central nervous system complications and immune correlates with one “type” of SLE might not be same as for others. This makes diagnosis and treatment challenging. Current therapies are largely centered on non-specific immunosuppressants, including corticosteroids, which can have profound long-term side effects. The development of biological therapies for SLE has proven problematic, not least the fact that different cytokines and cells might play different roles at different stages, in the many different manifestations. Key to developing new treatments for SLE will be unraveling the complex interplay between the many arms of the immune system, from the underlying inflammation, to the specific roles of the many cell types involved. Moreover, the ability to stratify patients in a way that tells us what shape their disease will likely take would help us to embrace, rather than grapple with, the heterogeneous nature of SLE, perhaps allowing us to develop different drugs for different subsets of patients.

This *Focus on Systemic Lupus Erythematosus* brings together reviews from some of the world’s leading experts on SLE and the immunology that underlies SLE pathogenesis. Mahajan et al. present the arguments for cell death and deficiencies in dead cell clearance by phagocytes as a central pathway in the pathogenesis of SLE. This review highlights findings that suggest phagocytes from patients with SLE may have a defect in their ability to engulf and clear apoptotic cells, leading to the exposure of autoantigens and a break in B cell tolerance, resulting in the development of autoimmunity and SLE.

The role of T cell signaling and different T cell subsets is the focus of the review by Rother and van der Vlag. In particular, they focus on aberrant T cell receptor (TCR) signaling and roles of Th17 and regulatory T cells (Tregs) in the development of SLE. Defects in the TCRζ chain, Syk kinase, and calcium signaling molecules, which have been associated with SLE, lead to the proliferation of autoreactive T cells, including Th17 cells. Concurrent with this is a reduction in numbers of Tregs and impairment of their function, leading to inappropriate and poorly controlled inflammation.

Cytokines play a complex and critical role in the pathoetiology of SLE. Lang et al. focus specifically on macrophage migration inhibitory factor (MIF) and its roles in SLE pathogenesis. MIF was first identified in the 1960s, yet still remains surprisingly enigmatic. There are numerous ways in which MIF might be linked with SLE, and this is borne out both in mouse models and clinical studies. Moreover, polymorphisms that lead to increased MIF secretion have been linked with SLE in interesting ways, potentially conferring protection against development of SLE, but leading to more severe disease after onset. The potential of targeting MIF therapeutically is also discussed.

Plasmacytoid dendritic cells (pDCs) have recently been demonstrated to play an important role in the development of autoantibodies and SLE pathogenesis. These cells are potent producers of type I interferons (IFN-I), a family of cytokines that has been intricately linked with SLE. The role of pDCs in SLE and other diseases has been difficult to establish due to their rarity, difficulty to identify, and rapid but transient release of IFN-I. Huang et al. review the most recent developments in this exciting area of SLE research.

Germinal centers (GCs) are key sites of B cell clonal expansion and affinity maturation. Also present in GC are follicular dendritic cells, which capture and retain antigen, T follicular helper cells, and T follicular regulatory cells. As Woods et al. discuss, many mouse models of lupus are characterized by the spontaneous formation of GC, induced through a number of different mechanisms, both innate and adaptive. The review also highlights the role of B cell-activating factor (BAFF), defects in dead cell clearance in GC, and the potential for targeting GCs therapeutically in SLE.

Kidney injury in SLE (lupus nephritis) is a major cause of both morbidity and mortality, affecting over half of all SLE sufferers over the course of the disease. Yung and Chan focus on the contribution of anti-double stranded DNA (dsDNA) antibodies to the pathology of lupus nephritis. Deposition of anti-dsDNA antibody-containing immune complexes in the kidney is an initiating factor in lupus nephritis. However, as this review discusses, direct and indirect binding of anti-dsDNA antibodies to cross-reactive antigens in the kidney also plays a major role. The downstream effects of this, including proliferation, apoptosis, inflammation, and fibrogenesis, are highlighted. In addition, recent data are discussed suggesting that mycophenolic acid (MPA), the active ingredient of the drug mycophenolate mofetil, has specific inhibitory effects on anti-dsDNA-induced processes, independent of its known immunosuppressive actions.

Finally, Gottschalk et al. provide an important overview of the current state of play with regard to SLE therapies and where they may lead in the future. Individual biological targets, especially cytokines, are discussed. In particular, the potential of targeting IL-6, an important inflammatory mediator in SLE, is highlighted, as well as the idea of targeting multiple pathways with combination treatments.

## Author Contributions

Both authors wrote and edited this editorial.

## Conflict of Interest Statement

The authors declare that the research was conducted in the absence of any commercial or financial relationships that could be construed as a potential conflict of interest.

